# Oral health status of inpatients with varying physical activity limitations in rehabilitation wards: A cross-sectional study

**DOI:** 10.1097/MD.0000000000026880

**Published:** 2021-08-13

**Authors:** So Jung Mun, Hyun Sun Jeon, Eun Sil Choi, Ree Lee, Sung Hoon Kim, Sun Young Han

**Affiliations:** a Department of Dental Hygiene, College of Software and Digital Healthcare Convergence, Yonsei University, Wonju, Republic of Korea; b Department of Dental Hygiene, Yeoju Institute of Technology, Yeoju, Republic of Korea; c Department of Rehabilitation Medicine, Yonsei University Wonju College of Medicine, Wonju, Republic of Korea.

**Keywords:** bedside oral exam, caries activity test, decayed, missing, filled teeth index, inpatient, modified Quigley-Hein plaque index, physical activity

## Abstract

Poor oral hygiene can be potentially life-threatening in inpatients. However, no basic protocol on oral hygiene customized for inpatients exists, and lack of oral care related knowledge, attitude, and skills among caregivers could be detrimental to the general health of patients. This study sought to identify the oral care practices and oral health status of inpatients with varying physical activity limitations in a rehabilitation ward.

Sixty-one inpatients in a rehabilitation ward were evaluated for their medical and physical conditions and oral health status. These were assessed using the bedside oral exam, decayed, missing, filled teeth index, plaque index, gingival index, and caries activity test.

In total, 40 men and 21 women (mean age, 56.6 years) were included in this study. Among them, 50.8% of the patients could brush their teeth unassisted, whereas 49.2% required assistance from an assistant for oral care. The proportion of patients receiving nasogastric tube feeding was higher in the group that could not provide oral self-care; 36.7% and 33.3% of these patients showed moderate and severe dysfunction, respectively, based on bedside oral exam. Scores for the swallowing, tongue, and total domains of bedside oral exam were poorer for patients who could not provide oral self-care (*P* < .01). The caries activity test indicated a moderate risk for both groups.

Our findings suggest that an oral care protocol that considers the physical activity limitations in inpatients in rehabilitation wards is necessary to minimize negative influences on the systemic health of these patients.

## 1 Introduction

Poor oral hygiene can be potentially life-threatening in case of inpatients. Infiltration of the lower respiratory tract, due to accumulation of bacteria and secretions in the oral cavity, and failure to maintain proper hygiene may lead to complications such as aspiration pneumonia.^[1]^ Dry mouth and plaque build-up within the oral cavity can cause dental caries, periodontal disease, and tooth loss, and patients with oral diseases have a higher incidence of aspiration pneumonia.^[2]^ Patients experience various problems during hospitalization, including increased plaque build-up, decreased salivary secretion, and increased risk of oral mucosal inflammation.^[3,4]^ Thus, assessing the oral health status of inpatients is essential.

Severely ill inpatients find oral care extremely difficult to maintain and usually tend to neglect it. During hospitalization, patients often find it difficult to practice proper oral hygiene on their own, due to physical and mental frailty and difficulty in obtaining the tools necessary for oral care.^[5]^ Keeping the mouth open for a long duration can easily lead to xerostomia, mucositis, and a shift from gram-positive to anaerobic gram-negative bacterial flora.^[3]^ Moreover, intubation or tube feeding makes maintenance of oral hygiene even more difficult, due to the presence of a tube in the oropharynx region.

The ability to maintain proper oral health by patients who need long-term hospitalization also affects their prognosis. Accumulation of dental plaque acts as a safe haven for respiratory pathogens, which increases the risk of aspiration pneumonia and associated mortality.^[6]^ Stroke patients often have dysphagia; therefore, they are often placed on long-term nasogastric (NG) tube feeding for non-invasive supply of nutrients and drug administration.^[7]^ However, nasal wing lesions, chronic sinusitis, gastroesophageal reflux, and aspiration pneumonia may occur easily in such patients and, thus, close attention is essential for the management of these patients, compared to those who are capable of eating on their own.^[8–10]^

Different oral care methods should be used according to the physical impairment experienced by patients during hospitalization; however, no such basic protocol or methods exist.^[11]^ Cerebrovascular disease, orthopedic disorders, and dementia are often accompanied by physical dysfunction, which make it difficult to perform self-oral care. Moreover, tube feeding and the ability to lift the head and upper body could render general oral care difficult. However, there are not enough health-care professionals in the wards to provide education about the proper and special oral care requirements of these patients. Assessments to ensure the proper oral health status identification and management of inpatients should be simple and objective. The bedside oral exam (BOE), which can be used to visually examine the oral health status of patients, was developed by Prendergast et al^[12]^ by modifying the Oral Assessment Guide developed by Eilers et al^[13]^ to assess the oral health status of intensive care patients. While checking for dental caries and dental plaque, various instruments can be used for examination, without needing an examination by a dentist. Qrayview (AIOBIO, Seoul, Republic of Korea) uses light in a range that is harmless to humans for visual inspection of early dental caries, dental plaque, and other oral diseases. It can be conveniently used for the oral examination of patients under long-term hospitalization because of its small size and portability. Irradiation with Qray light shows loss of fluorescence in case of demineralization due to dental caries, whereas areas with dental plaque build-up show red fluorescence.^[14,15,16]^ Cariview (AIOBIO, Seoul, Republic of Korea) is a caries activity test that can predict the potential risk level of dental caries using a simple method of collecting the saliva and dental plaque of patients with a sterile swab. Cariview is a tool that detects the risk of dental caries demineralized by acid by checking the total amount of acid production in the oral cavity using a mechanism similar to the Cariostat developed by Shimono and Sobue.^[17]^ Compared to Cariostat, Cariview has been developed to easily identify the risk of dental caries by changes in various color indicators based on the acid-producing ability of the bacteria in the oral cavity, which can be quantified through an optical analyzer.^[18]^ The use of different devices for the diagnosis of oral diseases in recent times has enabled us to reduce differences based on individual determination by dental professionals and to obtain objective results. Therefore, the aim of this study was to identify the differences in the oral health levels of inpatients based on the limitations in their physical activity and to identify factors that are vital to the effective planning of oral health care for inpatients.

## 2 Methods

### 2.1 Participants

This cross-sectional study was approved by the Institutional Review Board of Wonju College of Medicine, Yonsei University (ethical approval: YWDR-15-0118). All patients received sufficient explanations about this study and provided signed informed consent document. All patients consented to the use of data presented herein for publication.

All patients admitted to the Department of Rehabilitative Medicine in an advanced general hospital located in Gangwon Province, Republic of Korea, between March 6, 2017 and March 29, 2017, who consented to participate in this study were surveyed and underwent medical and oral health examinations.

The inclusion criteria for the study were as follows: age ≥20 years; had the ability to communicate independently or with the help of a legal guardian or caregiver; had the ability to open the mouth for oral health examinations. The exclusion criteria were as follows: age <20 years; unconscious status; edentulous patient. After an oral examination, the patients were taught an oral self-care method that considered a range of physical activity limitations expected to occur during hospitalization. The null hypothesis stated that there are no differences in oral health parameters between hospitalized patients who can provide oral self-care and those who rely on a third person for their oral care.

The present study was intended to compare the BOE score as a primary outcome according to the ability of the patients to perform oral self-care. Previous studies have indicated that the ability to perform oral self-care is an important factor in reducing oral disease and pneumonia risk. However, since BOE scores for patients able and unable to perform oral self-care had not been compared before, we calculated the number of samples.^[19,20]^ The number of patients per group (n = 34) was calculated after setting the level of significance at 5%, power of the study as 80%, and a hypothetical effect size of 0.7 using the G∗Power statistical software (Heinrich-Heine-Universität Düsseldorf, Düsseldorf, Germany), based on researcher discussion and pilot test.

### 2.2 Questionnaire

All patients completed a questionnaire consisting of 10 questions pertaining to the demographic characteristics (sex, age), medical conditions (duration of hospitalization, disease, feeding method), oral health behavior (ability to provide oral self-care or reliance on a third person for oral health care), and oral problems (pain, difficulty in chewing, dental caries, xerostomia, urgent need for dental treatment). The duration of hospitalization was classified into 4 groups; ≤10 days, 11 to 30 days, 31 to 60 days, ≥61 days. Disease was classified into 4 groups; brain disease, spinal damage, fracture and musculoskeletal disease, and amputation, cardiopulmonary disease, cancer, etc. The feeding method was divided into 2 groups; oral and others (NG feeding, percutaneous endoscopic gastrostomy feeding, and fasting). The questionnaire was developed by one of the researchers and was reviewed by a rehabilitation medicine specialist and 2 dental hygienists for validity to complete the final questionnaire.

### 2.3 Oral examination

An oral examination was performed by 2 dental hygienists who were trained to ensure high inter-examiner reliability. The intra-class correlation coefficient for the inter-examiner reliability was 0.782, which was in the good reliability range.

The BOE was used to visually assess the oral health status of patients by taking into consideration 8 domains: swallowing, lips, tongue, saliva, mucous membranes, gingiva, teeth, and odor. Each domain is assigned 1 to 3 points, and the total score is calculated by summing up the scores for each domain. The total score ranges from 8 (excellent oral health, normal) to 24 points (poor oral health).

The decayed, missing, filled teeth (DMFT) index and the plaque index (modified Quigley-Hein plaque index) were objectively assessed using the Qrayview.

Accurate gross examination while the patient is on a hospital bed is difficult to perform. To overcome this, a tooth was considered decayed if gray or brown fluorescence indicating mineral loss was detected on the occlusal surface by the Qrayview.^[21]^ In the assessment of filled teeth, defined as teeth with restorative treatment, sites of restorative treatment that were difficult to distinguish with the naked eye were easily detected by the Qrayview.^[22]^ From the 28 permanent teeth, lost teeth or teeth with only root stumps were classified as missing teeth.

Plaque accumulation was assessed using the modified Quigley-Hein plaque index,^[23]^ which is determined by the surface area of mature plaque observed with the Qrayview. A total of 6 teeth were assessed: maxillary right first molar, maxillary right lateral incisor, maxillary left first premolar, mandibular left first molar, mandibular left lateral incisor, and mandibular right first premolar.

The gingival index was evaluated for the same 6 teeth according to the method of Silness and Loe.^[24]^ Tooth mobility was assessed using a scale for rating visible tooth mobility.^[25]^ After the oral examination was complete, a caries activity test (Cariview) was performed according to the manufacturer's instructions.^[26]^ The total score ranged from 0 to 100, with scores of 0 to 40, 41 to 70, and 71 to 100 representing a low, moderate, and high risks, respectively.

### 2.4 Statistical analysis

The collected data were analyzed using SPSS Statistics version 23.0 (IBM, Chicago, IL), with the level of significance set at α = 0.05 (two-tailed). Among the 62 subjects who agreed to participate in the study during the study period, data from 61 participants were used for the final analysis, except for 1 who had insufficient questionnaire responses. Differences in the oral health status (BOE score, DMFT index, tooth mobility, remaining teeth, plaque index, gingival index, caries activity score) between patients who could perform oral self-care and those who could not were analyzed using an independent *t* test. The association between the ability to provide oral self-care and the feeding method was analyzed using a chi-square test. Multiple logistic regression analysis was performed to confirm the differences, and the influence of age and sex as a priori potential confounder among the variables was corrected.

## 3 Results

The demographic characteristics of the patients are demonstrated in Table 1. In total, 40 men and 21 women participated in this study. The mean age was 56.59 years, and the mean duration of hospitalization was 39.93 days. Brain disease was the most common cause of hospitalization (54.1%). Most patients could feed normally (68.9%), whereas, 31.1% to 24.6% of the feeding method (others) used NG tube feeding. Current oral problems were reported by 62.3% patients. The teeth were the most common site of oral problems (25.6%), followed by the gingiva (17.9%) and dentures (5.1%). The ability to perform oral self-care was observed for 50.8% patients, while 27.9% and 18.0% relied on a legal guardian and caregiver, respectively, for oral care; 3.3% patients could not receive oral care because of surgery.

**Table 1:** Demographic characteristics of inpatients with physical activity limitations in the rehabilitation ward.

Variable	
Sex	
Male	40 (65.6)
Female	21 (34.4)
Age group	
≤30 years	10 (16.4)
31–59 years	23 (37.7)
≥60 years	28 (45.9)
Duration of hospitalization	
≤10 days	13 (21.7)
11–30 days	20 (33.3)
31–60 days	16 (26.7)
≥61 days	11 (18.3)
Disease	
Brain disease	33 (54.1)
Spinal damage	11 (18.0)
Fracture and musculoskeletal disease	11 (18.0)
Amputation, cardiopulmonary disease, cancer, etc	6 (9.8)
Feeding method	
Oral	42 (68.9)
Others	19 (31.1)
Self-reported oral problems	
Yes	38 (62.3)
No	21 (34.4)
Not sure	2 (3.3)
Ability to perform oral self-care	
Yes	31 (50.8)
No	30 (49.2)
Total	61 (100.0)

Table 2 represents the comparisons of the demographic characteristics and the results of BOE and the caries activity test between patients who could perform oral self-care and those who could not. There were no differences between the 2 groups with regard to sex, duration of hospitalization, disease, and caries activity test results. However, the mean age (*P* = .02), proportion of patients who did not have an oral intake of food (*P* = .01), and proportion of patients with severe dysfunction according to BOE (*P* = .02) were significantly lower in the oral self-care group than in the group that could not perform oral self-care. When classified according to the diagnosis, as shown in Table 2, the rate of severe dysfunction in the group that could not perform oral self-care was 33.3%; this indicated that these patients had poor oral health.^[12]^

**Table 2:** Comparison of demographic characteristics, BOE, and caries activity test findings.

Variable	Total	Can perform oral self-care (*n*=61)	Cannot perform oral self-care	*P* value
Sex				.86
Male	40 (65.6)	20 (64.5)	20 (66.7)	
Female	21 (34.4)	11 (35.5)	10 (33.3)	
Age				.02
≤30 years	10 (16.4)	8 (25.8)	2 (6.7)	
31–59 years	23 (37.7)	14 (45.2)	9 (30.0)	
≥60 years	28 (45.9)	9 (29.0)	19 (63.3)	
Duration of hospitalization				.76
≤10 days	13 (21.7)	6 (19.4)	7 (24.1)	
11–30 days	20 (33.3)	12 (38.7)	8 (27.6)	
31–60 days	16 (26.7)	7 (22.6)	9 (31.0)	
≥61	11 (18.3)	6 (19.4)	5 (17.2)	
Disease				.38
Brain disease	33 (54.1)	16 (51.6)	17 (56.7)	
Spinal damage	11 (18.0)	4 (12.9)	7 (23.3)	
Fracture and musculoskeletal disease	11 (18.0)	8 (25.8)	3 (10.0)	
Amputation, cardiopulmonary disease, cancer, etc	6 (9.8)	3 (9.7)	3 (10.0)	
Feeding method				<.01
Oral	42 (68.9)	28 (90.3)	14 (46.7)	
Others	19 (31.1)	3 (9.7)	16 (53.3)	
BOE score				.02
Normal	26 (42.6)	17 (54.8)	9 (30.0)	
Moderate dysfunction	23 (37.7)	12 (38.7)	11 (36.7)	
Severe dysfunction	12 (19.7)	2 (6.5)	10 (33.3)	
Caries activity test				.76
Low risk	14 (24.6)	6 (20.7)	8 (28.6)	
Moderate risk	31 (54.4)	17 (58.6)	14 (50.0)	
Severe risk	12 (21.1)	6 (20.7)	6 (21.4)	

Table 3 represents the comparisons of the oral health parameters between the patients who could perform oral self-care and those who could not. Poorer scores for all domains of BOE were observed for the patients who could not perform oral self-care, with significant differences between groups in the scores for the swallowing, lips, tongue, and saliva domains. The mean BOE score was 13.2 for the patients who could not perform oral self-care, and 10.7 (normal range) for those who could; the score of the former group corresponded to moderate dysfunction (BOE, 11–14). The number of decayed teeth was smaller in the group that could not perform oral self-care (*P* = .40); however, the mean number of missing teeth in this group was 11.53, which indicated that 41% teeth were missing. The number of teeth that received restorative treatment was also smaller in this group than in the other group (*P* = .13). Results concerning tooth mobility, the number of remaining teeth, the amount of dental plaque, and gingival health were also poorer for the patients who could not perform oral self-care than for those who could, but the difference between the 2 groups was not statistically significant. In the caries activity test, both groups were classified as moderate risk.

**Table 3:** Comparison of oral health status parameters.

Variable	Total	Can perform oral self-care	Cannot perform oral self-care	*P* value
BOE score				
Swallowing	1.27 ± 0.58	1.06 ± 0.25	1.50 ± 0.73	<.01
Lips	1.39 ± 0.52	1.26 ± 0.45	1.53 ± 0.57	.04
Tongue	1.61 ± 0.75	1.29 ± 0.53	1.90 ± 0.80	<.01
Saliva	1.56 ± 0.64	1.35 ± 0.49	1.77 ± 0.73	.01
Mucous membranes	1.23 ± 0.42	1.13 ± 0.34	1.33 ± 0.48	.06
Gingiva	1.47 ± 0.54	1.39 ± 0.56	1.53 ± 0.51	.29
Teeth, dentures	1.74 ± 0.77	1.61 ± 0.72	1.83 ± 0.79	.26
Odor	1.69 ± 0.62	1.58 ± 0.62	1.80 ± 0.61	.17
Total	11.7 ± 3.03	10.68 ± 2.26	13.20 ± 3.22	<.01
DMFT index				
DT	1.03 ± 1.39	1.13 ± 1.28	0.83 ± 1.42	.40
MT	9.40 ± 10.26	7.61 ± 10.48	11.53 ± 9.87	.14
FT	3.95 ± 4.19	4.61 ± 4.53	3.00 ± 3.48	.13
DMFT	14.39 ± 9.46	13.35 ± 8.88	15.37 ± 10.21	.41
Tooth mobility	0.65 ± 1.37	0.39 ± 0.76	0.87 ± 1.78	.18
Remaining teeth	18.56 ± 10.27	20.35 ± 10.46	16.43 ± 9.90	.14
Plaque index	1.14 ± 0.95	0.90 ± 0.74	1.40 ± 1.09	.05
Gingival index	0.73 ± 0.59	0.58 ± 0.52	0.87 ± 0.65	.07
Caries activity score	56.82 ± 15.91	58.10 ± 15.63	54.75 ± 16.07	.43

Table 4 represents the influence of oral health status parameters on oral self-care after adjustment of age and sex as confounding factors. The oral health status parameters were selected from Tables 2 and 3, which were significant in the chi-square test. The significance probability for the Hosmer and Lemeshow test was 0.983, which was an appropriate model, and Nagelkerke R squared showed an explanatory power of 0.355. Consequently, the feeding method (others) group had a 6.53 times higher risk of not being able to perform oral self-care (*P* = .01), compared to those who were fed orally; moreover, the dryness of the tongue was directly proportional to the odds ratio by 2.84 times (*P* = .04).

**Table 4:** Influence of oral health status parameters on the group that could not perform oral self-care according to logistic regression analysis.

	Oral self-care	
Variable	OR (95% CI)	*P* value
Feeding method		.01
Oral	1.00 (reference)	
Others	6.53 (1.49–28.65)	
BOE: tongue	2.84 (1.07–7.51)	.04
Cox & Snell R^2^: 0.266	Nagelkerke R^2^: 0.355	

## 4 Discussion

The present study was conducted to investigate the association between the ability to perform oral self-care and the oral health status in inpatients with physical activity limitations in a rehabilitation ward. We conducted a questionnaire survey to investigate the medical conditions and status of oral health care as well as oral health examinations to assess the oral health status of the patients. We found that 49.2% patients were incapable of providing oral self-care, and 33.3% of these patients had severe dysfunction according to the results of BOE. These patients also showed poorer scores for the swallowing, lips, tongue, and saliva domains of BOE and a greater amount of dental plaque than did the patients who could perform oral self-care.

Oral health care is one of the most important interventions that can lower the risk of hospital-acquired infections among inpatients, and it is reported to decrease the duration of hospital stay and mortality rate and improve the nutritional status.^[27–29]^ However, oral health care is not efficiently provided because of the lack of education and awareness about oral health management among nurses in charge of the oral health of inpatients, lack of resources or protocols, and the relatively low importance of oral health care when compared with other medical tasks.^[11,30,31]^ In a recent study involving nurses working in general hospitals in Republic of Korea, 39.2% respondents provided oral care to their patients.^[32]^ Of these respondents, those who worked in intensive care units exhibited an oral care practice rate of 83.9%, whereas those working in general wards exhibited an oral care practice rate of 15.1%.^[32]^ Factors that hindered oral care provision by nurses included insufficient time (51.8%), low priority given to oral care (31.9%), and lack of knowledge (6.4%). Thus, oral care was not adequately provided by nurses in Republic of Korea.^[32]^

Inpatients with physical disabilities and impaired body control become dependent on others for oral care. Those with difficulty in swallowing have several constraints on oral care activities, even when they are performed by others. The oral hygiene of patients who rely on a third person for oral care can vary greatly depending on the level of knowledge and attitude of the individual providing oral care. Therefore, it is necessary to educate these providers on oral care methods that take into consideration patients’ physical activity limitations. A previous study reported that independence for oral health care was significantly associated with the oral health status (Oral Health Assessment Tool) of patients with stroke.^[33]^ Another study reported significant differences in the number of methicillin-resistant and methicillin-sensitive *Staphylococcus aureus* colonies after patients with stroke received oral care from nurses who were professionally educated about daily oral care.^[34]^ In patients who have undergone stroke, the use of an electric toothbrush with chlorhexidine has been shown to be more effective in reducing plaque and gingival bleeding scores, and decreasing the number of *S aureus*, compared with a manual toothbrush and toothpaste.^[35,36]^ Providing oral care to patients is different from providing normal oral care. As such, it is not only necessary to select oral care products that are easy to apply, reduce the risk of aspiration, and use antimicrobial agents, but also to personalize them such as for them to best suit each patient's physical situation.

In general wards, oral care may be administered to make the patient comfortable and for hygiene purposes, whereas it is administered in intensive care units for infection prevention.^[34]^ Therefore, nurses in general wards provide lower level of oral care than those in intensive care units.^[37,38]^ In a questionnaire survey conducted in a stroke unit in Scotland, only 21% nurses in the unit followed the oral care protocol, whereas only 23% used an oral care assessment tool to assess the oral health conditions of patients.^[11]^ In a Korean survey, only 15.1% nurses in hospital wards were found to provide oral care.^[32]^

A significant number of patients with stroke experience difficulty in swallowing^[39]^ and xerostomia.^[40]^ These patients are at an increased risk of aspiration pneumonia, dental caries, and periodontal diseases due to increased plaque accumulation resulting from the lack of oral care. In the present study, 45.9% patients were aged ≥60 years and exhibited ongoing deterioration of oral function. In addition, more than 50% patients were hospitalized for brain disease and were in a physically constrained situation where they could not manage their own oral health. According to BOE, patients who could not perform oral self-care showed a poor oral health status, with significantly high scores for swallowing impairment, dry lips, tongue cracks, and lack of saliva (Table 3). Particularly, tongue health showed a strong influence on oral self-care even after adjusting for age and sex (Table 4). If these symptoms continue, reduced amount of self-cleaning due to inadequate saliva can induce xerostomia and increase the risk of aspiration pneumonia.

The amount of dental plaque deposition was higher in patients who could not perform oral self-care, but the difference was not statistically significant (Table 3). The Qrayview can detect mature plaque; thus, it provides results that are clinically more important than those obtained by coloring agents that also detect immature plaque. Han et al^[14]^ have reported a higher amount of *Prevotella intermedia* and *Streptococcus anginosus* in plaque detected by the quantitative light-induced fluorescence than in those that were not detected by the device. Another study reported that plaques detected by the quantitative light-induced fluorescence showed a higher proportion of anaerobic bacteria.^[41]^

In a study that investigated changes in oral health behaviors before and after hospitalization, patients who were unable to brush their own teeth were found to be hospitalized for over a month, hospitalized in a ward in a neurology or neurosurgery department, and/or aged ≥50 years.^[42]^ Focusing treatment on the affected site or the site of damage during the hospitalization period can lead to the development of a disease in other healthy parts of the body or degenerative changes in an organ. To prevent this, it is necessary to perform appropriate assessments, provide appropriate education and equipment, and train oral care providers (legal guardians, caregivers, nurses) if necessary. All these processes must be conducted at the individual level.^[43]^ In the present study, the oral health status differed between patients who could provide oral self-care and those who could not. This means that patients who can perform physical activities during hospitalization can perform oral self-care as well as they could before hospitalization. Although the present study did not investigate changes in oral health behaviors at the time of hospital admission, a previous study reported a decreased frequency of tooth-brushing and lesser use of oral care products following hospital admission.^[42]^

Although education about oral care methods alone can be effective for patients without physical activity limitations, special considerations are required for patients who cannot perform oral self-care because of physical activity limitations. In the present study, the number of patients who received NG feeding was higher in the group that could not perform oral self-care (N = 13 and 2) (data not provided), probably because legal guardians or caregivers in charge of providing oral care tend to find it unnecessary to care for their patient's oral health because they are not taking food via the mouth. Although we did not investigate how oral care was being provided by the third person, it was generally observed that patients who were admitted to their ward immediately after surgery failed to fully clean their mouth using a foam swab or distilled water without a toothbrush.

This study has some limitations. First, the number of samples was small, there may be a selection bias by recruiting only from 1 institution, and there is a limit to generalizing the results to other populations. This study was carried out as an observational study conducted on patients admitted to the rehabilitation department of 1 general hospital for a certain period. Therefore, the original goal of recruiting 34 subjects per group was not completely achieved. Although there were limitations due to the size of the hospital ward and a short investigation period, we have confirmed that patient independence may be an important factor influencing oral health. In addition, the difference in effect size according to the BOE score, after the end of the study, was calculated to be 0.9, which is higher than the investigator's prediction of 0.7. Second, patients were not questioned about their own methods of oral care because the aim was to investigate the oral health status of inpatients. However, a basic understanding of the methods used by the patients could be obtained after educating them about oral care methods that considered their physical activity limitations. Future studies must consider oral health behaviors during hospitalization. Third, plaque and gingival examinations were performed only for a few selected teeth; hence, the findings do not reflect the overall health status of a patient. However, the examined teeth were selected on the basis of index teeth used in previous studies. Therefore, our tooth selection may not have significantly influenced the results.

This study analyzed oral health parameters for inpatients with varying physical activity limitations who were admitted to a rehabilitation ward in a hospital. Despite the study limitations, the findings confirm that the abilities of individuals in charge of providing oral care (legal guardian, caregiver, nurse) to a patient who is unable to manage his/her own oral health should be assessed at the individual level, and these people should be motivated to instruct their patients on appropriate tooth cleaning techniques. Moreover, efforts to use special oral care tools are necessary. The results of this study may be used as a foundation for the development of oral care methods specific to inpatients. In the long term, they may contribute to the development of an oral care protocol that considers the physical activity limitations of inpatients.

## Acknowledgments

We would like to thank Editage (www.editage.co.kr) for English language editing. We would also like to express our gratitude to Yunhee Kim for supervising the statistical analysis and interpretation of the results of this study.

## Author contributions

SJ designed the questionnaire and wrote the draft. HS participated in research design and writing the draft. ES and RL participated in data collection. SH contributed to the design of the questionnaire and the investigation of clinical patients. SY participated in the oral examinations of patients, analyzed data, and reviewed the overall study. All authors read and approved the final manuscript.

**Conceptualization:** So Jung Mun, Hyun Sun Jeon, Sun Young Han.

**Data curation:** Sung Hoon Kim, Sun Young Han.

**Formal analysis:** Sun Young Han.

**Funding acquisition:** So Jung Mun, Sun Young Han.

**Investigation:** Eun Sil Choi, Ree Lee.

**Methodology:** Hyun Sun Jeon, Sung Hoon Kim.

**Project administration:** So Jung Mun.

**Resources:** Sung Hoon Kim.

**Software:** Sun Young Han.

**Supervision:** Sun Young Han.

**Validation:** So Jung Mun, Sun Young Han.

**Visualization:** Eun Sil Choi.

**Writing – original draft:** Hyun Sun Jeon, Sun Young Han.

**Writing – review & editing:** So Jung Mun, Eun Sil Choi, Ree Lee, Sung Hoon Kim.
